# Quality Improvement Initiative to Improve Healthcare Providers’ Attitudes towards Mothers with Opioid Use Disorder

**DOI:** 10.1097/pq9.0000000000000453

**Published:** 2021-08-26

**Authors:** Susan Ford, Leslie Clarke, Michele C. Walsh, Pierce Kuhnell, Maurizio Macaluso, Moira Crowley, Richard McClead, Scott Wexelblatt, Carole Lannon, Heather C. Kaplan

**Affiliations:** From the * Department of Pediatrics, Division of Neonatology, Rainbow Babies and Children’s Hospital, Cleveland, Ohio; † Department of Pediatrics, Case Western Reserve University, Cleveland, Ohio; ‡ Division of Biostatistics and Epidemiology, Cincinnati Children’s Medical Center, Cincinnati, Ohio; § James M. Anderson Center for Health Systems Excellence, Cincinnati Children’s Hospital Medical Center, Cincinnati, Ohio; ¶ Department of Pediatrics, University of Cincinnati, College of Medicine, Cincinnati, Ohio; ∥ Department of Pediatrics, Nationwide Children’s Hospital, Columbus, Ohio; ** Department of Pediatrics, Ohio State University, Columbus, Ohio; †† Perinatal Institute, Cincinnati Children’s Hospital Medical Center, Cincinnati, Ohio

## Abstract

**Introduction:**

Individuals with opioid use disorder often report feelings of shame and describe feeling judged negatively. These feelings are especially true for pregnant women with opioid use disorder. The Ohio Perinatal Quality Collaborative conducted a multimodal quality improvement initiative for infants born with Neonatal Abstinence Syndrome (NAS). An important component of the project was focused on improving staff attitudes toward mothers of infants with NAS.

**Methods:**

The Ohio Perinatal Quality Collaborative implemented an education program for healthcare providers at 39 participating hospital units regarding opioid use as a chronic disease and principles of nonjudgmental, trauma-informed care. Healthcare providers partnered with the mother of infants with NAS in the care of the infant and connected with local community resources. This work was a subcomponent of an overall multimodal quality improvement project. Healthcare provider attitudes were measured with the “Attitude Measurement: Brief Scales” questionnaire anonymously, at 3 different time points throughout the project. Attitude change was measured by pretraining and posttraining scores. ANOVA methods were used to compare individual items and a summary score across the 3 surveys.

**Results:**

Summary scores improved significantly from 18.99 at baseline (January–March 2014) to 19.94 (*P* < 0.0001) in February 2015 and were maintained at 20.05 in July 2016.

**Conclusions:**

A nonjudgmental attitude toward mothers of infants with NAS is an important component of compassionate care. Improving healthcare provider attitudes can benefit a mother of an infant with NAS and help preserve the mother–infant dyad.

## Abstract 2

Supplemental Digital Content is available in the text.

## INTRODUCTION

There has been a dramatic increase in Neonatal Abstinence Syndrome (NAS) following an infant’s in utero exposure to opioids.^1^ In 2016, 6% of pregnant women in the United States reported illicit drug use.^2^ National rates of opioid use disorder (OUD) at delivery have more than quadrupled during 1999–2014.^3^ At the state level in Ohio, mothers diagnosed with drug dependence at delivery have increased dramatically from 302 cases in 2006 to 1,967 in 2018. Respectively, the hospital discharge rates for infants diagnosed with NAS have soared from just 305 in 2006 to 1,932 in 2018.^4^

With a goal of collaboratively improving care for mothers and their infant with NAS, in January 2014, the Ohio Perinatal Quality Collaborative (OPQC) began work with participating Level II and III Neonatal Intensive Care Units and newborn nurseries across the state. The overall SMART aim of the NAS project was to “increase identification of and compassionate withdrawal treatment for full-term infants born with NAS and reduce the length of stay by 20% across participating sites by June 30, 2015.” The collaborative successfully decreased both lengths of opioid treatment and hospitalization by 10%, resulting in over 2,000 fewer days of in-hospital care.^5^ An intervention listed within the key driver diagram addressed the shame and stigma associated with opioid use, especially during pregnancy: “improve recognition and non-judgmental support for narcotic addicted women and infants.” Mothers have described feeling judged or condemned for using opiates while pregnant.^6^ Previous studies suggest that healthcare providers’ attitudes towards both illicit drugs and those with OUD may affect the provision of care for this population.^7^ With the impact trauma can have on the health of an individual, it is important for healthcare organizations to include a trauma-informed approach to care.^8^

The goal of this arm of the project was to improve healthcare providers’ attitudes toward mothers of infants with NAS. To improve care and support of the mother–infant dyad, OPQC chose to promote a nonjudgmental and compassionate approach toward the mother with OUD through an educational intervention. In this report, we describe the effects of the OPQC intervention targeting attitudes among participating NICU healthcare providers.

## METHODS

OPQC conducted a multisite QI collaborative beginning in January 2014 and continuing through September 2016. Twenty-six of 26 (100%) Level III NICUs, 26 of 28 (93%) Level II Special Care Nurseries, and 2 Level 1 Newborn Nurseries who treated infants with NAS participated. Thirty-nine of these sites were used for this data analysis as they met the minimum requirement of five or more questionnaire responses at all time points. Multidisciplinary teams, which included a physician, nurse, social worker, pharmacist, dietician, and addiction specialist, were formed to drive the initiative at each site.

Efforts to improve the nonjudgmental care of women with OUD were included as part of an overall initiative to improve care delivery for infants with in utero opiate exposure. The OPQC NAS driver diagram, which detailed the overall theory of change, explicitly acknowledged the role of changing healthcare provider attitudes toward mothers of infants with NAS (**see Supplemental Digital Content 1,** which describes OPQC. NAS Project Key Driver Diagram. Available at: https://www.opqc.net/nas-kdd, http://links.lww.com/PQ9/A291). The project protocol was considered QI and not human subjects research by the institutional review board of the Cincinnati Children’s Hospital Medical Center.

An adapted Breakthrough Series model, developed by the Institute for Healthcare Improvement, was used for this collaborative project.^9^ Utilizing this model, participating teams attended monthly action period calls and in-person learning sessions twice annually where topics regarding compassionate non judgmental care and addiction as a chronic disease were highlighted. In an “all teach, all learn” approach, teams collaboratively shared their work on improving healthcare provider attitudes throughout the project. Planned tests of change—PDSAs to plan, test, study, act—were used as a model of improvement method to bring about change, standardization, and measured improvement. Team PDSAs regarding the driver of improved support for the mom with OUD were emphasized and shared on action period calls and in-person learning sessions (**see Supplemental Digital Content 2,** which describes OPQC (2018). NAS Project Action and Sustain Period Calls. Available at: https://www.opqc.net/nas-period-calls, http://links.lww.com/PQ9/A292). Monthly action period webinars and learning session content from the NAS project are accessible on the OPQC website.^10^ A listing of educational interventions accompanied with team PDSAs explicitly focused on improving nonjudgmental care of the mother with OUD is detailed in Table 3.

### Interventions

Interventions designed to improve recognition and nonjudgmental support of narcotic-exposed women and their infants were tied to 3 key areas. These areas included: education of healthcare providers, a better partnership between the healthcare providers and the mothers, and linkages to community resources to better support the mother–infant dyad. Specific interventions were included as follows.

### Education of Healthcare Providers

An addiction specialist/family practice physician spoke at the initial learning session regarding addiction as a chronic disease. This session highlighted the pathophysiology and chronicity of addiction, the benefit of medication-assisted treatment throughout pregnancy, and the philosophy of trauma-informed care. The stigma associated with drug use was also discussed in detail. The inclusion of an addiction Specialist in each participating site’s NAS QI team was strongly encouraged.

Unit-wide educational training for all NICU healthcare providers about living with OUD occurred as well. OPQC purchased a video created by the Vermont Oxford Network entitled “Nurture the Mother-Nurture the Child” for each participating hospital.^11^ The video describes how attitudes and stigma toward patients with addiction can profoundly impact a mother and can be a barrier to her recovery from addiction. Hospital teams distributed the video widely to their entire staff by uploading it onto the hospitals’ local learning management systems.

### Improved Partnerships between Healthcare Providers and Mothers

Exposure to stories of pregnant women with OUD through a parent panel discussion at a learning session allowed healthcare providers to hear first-hand accounts from mothers of infants with NAS. Many described feeling excluded in the care of their hospitalized infant. Partnering with the mother in the use of the Finnegan scoring tool was advised, as was breastfeeding, when appropriate. After an action period call discussing the benefit of breastfeeding, teams were instructed to review their hospital policy regarding its use for the substance-exposed infant. Many teams updated their policies and shared these practices on a follow-up action period call. Teams were also encouraged to test rooming-in for the infant with the mother if space allowed. One team tested moving the infant’s site of care to their pediatric unit when rooming-in was not an option in their NICU step-down unit.

### Linkages to Community Resources

Participating hospital teams reached out to regional community medication-assisted treatment programs and support organizations to determine available resources to support the mother–infant dyad. The teams were also asked to identify barriers or challenges in accessing the resources. Additionally, each site was encouraged to interact with at least 1 organization and share with other sites their findings in a PDSA manner utilizing a standardized storyboard at a learning session.

### Measures

**Fig. 1.**
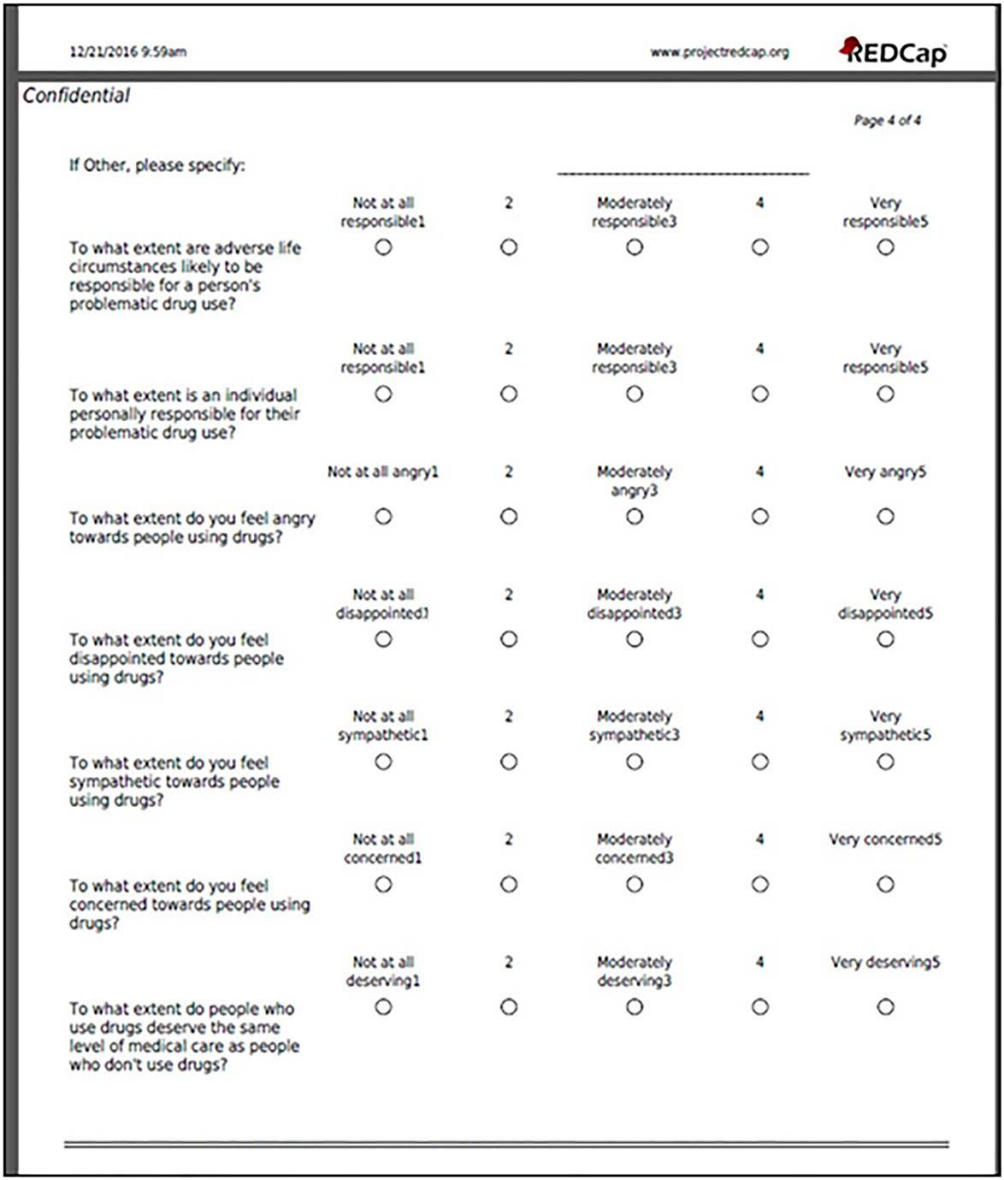
Attitude measurement: brief scales questionnaire. Adapted and reprinted with permission from the Australian Research Centre on AOD Workforce Development, 2006.

OPQC measured attitudes with a brief anonymous and voluntary questionnaire to each participating site. The questionnaire was a modification of the National Centre for Education and Training on Addiction’s “Attitude Measurement: Brief Scales” included in the “Health Professionals’ Attitudes Towards Licit and Illicit Drug Users Training Resource.”^12^ It included 7 questions graded on a 5-point Likert scale (Fig. 1). OPQC modified the “Attitude Measurement: Brief Scales” questionnaire to exclude one question deemed to have substantial overlap with another.

All attending and fellow physicians, neonatal and pediatric nurse practitioners, registered nurses, and social workers working in the participating neonatal hospital sites were asked to complete the questionnaire. Teams were notified of the questionnaire at three time points—baseline, mid-collaborative, and near the end of the collaborative. Baseline questionnaires were sent out between January and March 2014. All sites received the questionnaire again in February 2015 and July 2016.

Data were collected in REDCap, a HIPAA compliant electronic data tool hosted at Cincinnati Children’s Hospital Medical Center.^13,14^ REDCap was used to send the email questionnaire with the capability to provide 1 hyperlink that could be submitted completely anonymously by any number of respondents at each participating hospital. Team key contacts and lead physicians were recruited via email and encouraged on the subsequent action period call to share the hyperlink with the healthcare providers in their hospital unit. Reminders were sent out accompanied with the directions for completion at three time periods: 2 weeks before the submission deadline, and again at 1 week and the day before closing the RedCap questionnaire. Because the data were submitted anonymously, OPQC was unable to link individual respondents across the time points.

### Statistical Analysis

Each of the 7 items was individually examined and also scored using a calculated summary scale. Because responses to the questions could go in opposite directions (ie, higher scores meant better responses in some questions and worse responses in other questions), the scores were recorded to reflect the same direction of change, and the recoded values were summed to compute the summary scale. Proc Corr in SAS 9.4 (SAS Institute, Cary, N.C.) was used to assess internal consistency of the responses to the questionnaire and found a set of 6 questions that yielded a Cronbach’s alpha value of 0.7. A sum of those 6 questions was used as a summary scale.

To examine changes in attitudes over time for the collaborative as a whole, we used mixed regression models with random hospital effects and estimated hospital-specific mean responses (scores) for each of the 7 Attitude Measurement: Brief Scales questions and the summary score at each time point. Inclusion criteria included a site submission of at least 5 questionnaires at all 3 time points. All models were computed using Proc Mixed in SAS 9.4 and included the hospital-specific mean score as the dependent variable, time point, and count of questionnaire responses as fixed effects, and hospital as a random effect. OPQC data analysts then evaluated differences between mean scores at different time points using the Tukey–Kramer method to adjust for multiple comparisons due to unequal sample sizes among the time points.

Evaluation of the level of change for each site was measured by 2 different methods. The first step included calculating the hospital and time point-specific least-squares mean scores for each question and the summary scale. The analysts then evaluated within-site differences in the least-squares means between time points using the Tukey–Kramer method to adjust for multiple comparisons (see above). They then determined a positive linear trend across all time points for each of the 7 questions and the summary scale by evaluating linear contrasts.

## RESULTS

Responses were received from 1,496 individuals during the baseline phase, 1,684 individuals at the mid-point, and 1,898 individuals during the final assessment. The respondents who participated are described in Table 1. A vast majority of the respondents were nurses. Unit social workers were included in the survey for time points 2 and 3.

**Table 1.** Questionnaire Respondents by Role

	Baseline, n = 1,496, 39 Hospitals	February 2015, n = 1,684, 39 Hospitals	July 2016, n = 1,898, 39 Hospitals
Attending MD	105 (7%)	117 (7%)	138 (7%)
Fellow	19 (1%)	17 (1%)	22 (1%)
Neonatal nurse practitioner	101 (7%)	96 (6%)	102 (5%)
Registered nurse	1,194 (80%)	1,292 (77%)	1,429 (75%)
Social worker	0 (0%)	21 (1%)	33 (2%)
Other	76 (5%)	141 (8%)	171 (9%)

May exceed 100% due to rounding.

**Fig. 2.**
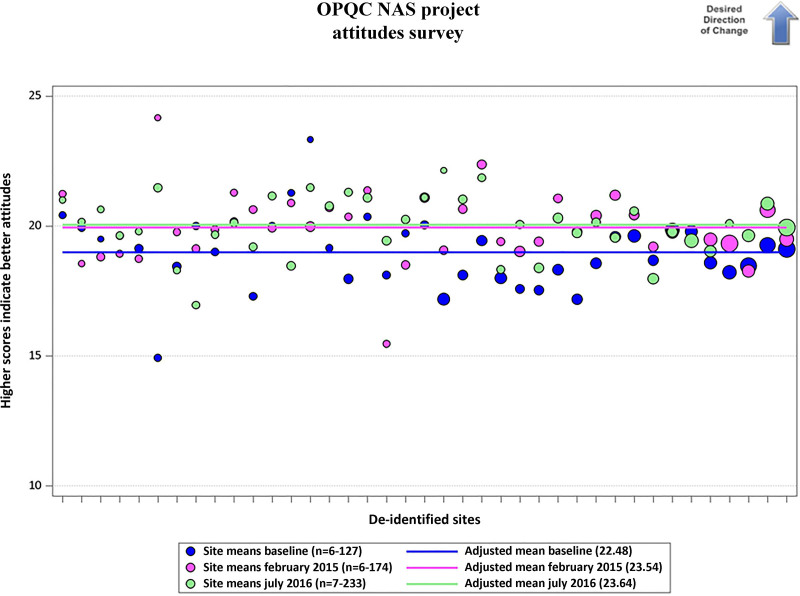
Attitude measurement survey sum of scores.

Healthcare providers’ attitudes improved and maintained improvement throughout the different time points. Results are provided for each question in Table 2 and Figure 2. Five of the 7 questions showed statistically significant improvements in healthcare providers’ attitudes on the 5-point Likert scale (**see Supplemental Digital Contents 3–7,** which describes Bubble plot—to what extent do you feel angry towards people using drugs? To what extent is an individual personally responsible for their problematic drug use? To what extent do you feel disappointed toward people using drugs? To what extent do you feel sympathetic toward people using drugs? To what extent do people who use drugs deserve the same level of medical as people do not use drugs? http://links.lww.com/PQ9/A293). Healthcare providers responding to the questionnaire reported a decrease from baseline to the February 2015 questionnaire in the extent to which they feel angry towards people using drugs (2.41–2.27, *P* = 0.02); the extent to which they felt the individual person is responsible for their drug use (4.21–4.01, *P* ≤ 0.0001); the extent to which they felt disappointed in people using drugs (3.11–2.92, *P* = 0.01); and an increase in the extent to which they feel sympathetic to people using drugs (2.95–3.13, *P* = 0.002). These significant changes persisted through the July 2016 questionnaire.

**Table 2.** Change in Score Over Time for Each Attitude Question

	Desired Direction of Change	Adjusted Mean (95% CI) Baseline	Adjusted Mean (95% CI) February 2015	Adjusted Mean (95% CI) July 2016
To what extent do you feel angry towards people using drugs?	Down	2.41 (2.31–2.50)	2.27 (2.18–2.37)*	2.29 (2.19–2.38)*
To what extent is an individual personally responsible for their problematic drug use?	Down	4.21 (4.13–4.28)	4.02 (3.94–4.09)*	3.98 (3.90–4.05)*
To what extent do you feel disappointed toward people using drugs?	Down	3.11 (3.00–3.22)	2.92 (2.81–3.03)*	2.95 (2.85–3.07)*
To what extent are adverse life circumstances likely to be responsible for a person’s problematic drug use?	Up	3.65 (3.57–3.72)	3.71 (3.64–3.78)	3.72 (3.65–3.79)
To what extent do you feel sympathetic toward people using drugs?	Up	2.95 (2.85–3.04)	3.13 (3.03–3.23)*	3.14 (3.04–3.24)*
To what extent do people who use drugs deserve the same level of medical care as people who do not use drugs?	Up	4.49 (4.43–4.55)	4.56 (4.50–4.62)	4.57 (4.50–4.63)*
To what extent do you feel concerned towards people using drugs?	Up	4.15 (4.08–4.22)	4.13 (4.06–4.20)	4.19 (4.12–4.26)
Sum of Scores Summary Scale	Up	18.99 (18.57–19.40)	19.94 (19.53–20.36)*	20.05 (19.63–20.46)*

*Denotes a significant (*P* < 0.05) difference from the mean at baseline after adjusting for site and multiple comparisons.

**Table 3.** Interventions to Support Nonjudgmental Care

Category	Intervention	Timing
Addiction as a chronic disease	• OPQC kick off learning session presentation	• January 2014
	• OPQC onboarding webinar presentation	• March 2014
	• Suggestion for inclusion of addiction specialist on internal NAS project team	• May 2014
	• Unit staff meetings with open discussion of personal bias	• April 2015
	• Presentation from participating hospital on “NAS Volunteer Specialist Training” to include nonjudgmental approach and the topic of addiction as a chronic disease	• June 2015
Vermont Oxford Network DVD “Nurture the Mother-Nurture the Baby”	• Sites upload DVD to internal Learning Management Systems (and include in unit orientation curriculum)	• January–December 2014
	• DVD viewing at Lunch and Learn with staff	• May 2014
	• Extend DVD to Normal Newborn staff	• October 2014
	• Extend DVD to L&D staff	• December 2014
Improved partnership between health care providers and mothers of infants with NAS	• Suggestion for scheduled maternal prenatal consults with neonatology (at approximately 34 wks gestation)	• June 2014
	• Collaboration with mother and RN in Finnegan scoring	• June 2014
	• Led to a mom being invited to parent advisory committee	
	• Site follow-up on surveys to mothers of NAS infants postdischarge regarding their experience in NICU	• October 2014
	• Parent panel on life experiences at January Learning Session	• January 2015
	• Parental (mother) input at bedside rounds	• June 2015
	• Video option for input when unable to attend in person	
	• Suggestion of “MOMS Daily Update”—daily update completed at bedside	• July 2016
Linkages to community resources	• Outreach to local residential treatment center for women	• January 2015
	• Suggestion of prenatal site visits for mothers with OUD	• January 2015
	• Suggestion of prenatal classes regarding NAS education and care	• January 2015

Additionally, a significant increase was noted from baseline to July 2016 for the extent that questionnaire respondents felt life circumstances were responsible for drug use (4.49–4.57, *P* = 0.04). Though the respondent pool changed slightly over time with the inclusion of social workers at time points 2 and 3, we do not believe the primary driver of the change in attitudes is related to this modification. Regression models were studied removing social workers from the model, and the results were found to be the same as previous models: significant improvement in attitudes remained.

The summary scale showed a significant increase between baseline and February 2015 (18.99–19.94, *P* < 0.0001), and this increase was maintained between February 2015 and July 2016. Summary scale results are provided in Table 2 and Figure 2.

## DISCUSSION

Clinical caretakers often have judgmental responses to individuals with OUDs.^15–18^ Persons with OUDs report that the stigma associated with such use interferes with their desire to seek and remain in treatment.^19,20^ In this study, OPQC reports the results of a trauma-informed standardized educational program that included presentations by mothers in recovery from OUD and healthcare providers partnering with the mother in the care of her infant that contributed to overall improvements in health attitudes across a large statewide quality collaborative focused on the care of mothers and infants with opioid exposure. We knew that healthcare professionals’ attitudes toward mothers of NAS infants were variable. Some had such a judgmental stance that it could interfere with creating a welcoming environment and delivering the nonpharmacological bundle. We did not know whether the educational tools we had developed to address this problem were effective. We used a simple measurement tool that we thought was appropriate for the situation and found that the educational intervention moved the dial and produced a statistically significant positive change.

The improvements to attitude scores were maintained throughout the collaborative. Moreover, there were no instances where a change to a more positive attitude at an intermediate time point reverted to a less positive attitude later. We believe that the scores changed between time points 1 and 2 when most of our educational interventions took place, and the scores were maintained between time points 2 and 3 as fewer interventions were done during that time. To our knowledge, this is the first assessment of the impact of this standardized curriculum to improve attitudes.

There are several reasons why the OPQC curriculum may have improved care provider’s attitudes. Guided by the principles of learning from top performances—a Safety II approach of highly reliable organizations—we informally spoke with nursing leaders at participating hospital units with the most significant attitude improvements to gain awareness on how those changes may have occurred. Nursing leaders at these hospitals cited three areas as critical components leading to attitude change: leadership support and engagement; a learning environment that emphasized multidisciplinary education with ongoing mandatory classes; and partnering with parents, including increased interaction with mothers, actively engaging them in their infant’s care.

OPQCs work has similar findings to those conducted in other settings. Clarke and colleagues found in a systematic review of published reports from emergency room physicians and nurses an overall negative attitude to those with OUDs.^21^ Other studies have evaluated the impact of education on nursing and medical students, and residents with mixed results.^15,17,18^ Some studies have found that the intensity of the teaching experience and exposure to vignettes about persons with OUD were effective in changing providers attitudes.^22^ Less is known about influencing the attitudes of practitioners who are well established in their careers. Englander et al^16^ found that education using a chronic illness model and emphasis of the disorder as a mental illness rather than a moral failing was successful in changing hospital providers’ attitudes.

### Limitations

This work has some limitations. The “Attitude Measurement: Brief Scales” questionnaire was not previously tested for reliability or validity. OPQC also acknowledges the limitation of not being able to track individual respondents across the time periods due to the questionnaire’s anonymous nature. There is also a possibility of voluntary participation response bias as there was no way to determine the participation rate. Also, it is possible that the changes in attitudes seen were not directly related to the educational curriculum but other concurrent changes in these hospital sites.

It is also challenging to address the significance or magnitude of the improvement. We cannot demonstrate that the change in attitudes produced by the intervention was sufficient to meaningfully increase “hard” outcome indicators (eg, length of stay), but speculate that even a small change in the right direction can improve the care environment in ways that are not easily measured. We recognize the need for further developing the theory and the measurement techniques, perhaps by adding an instrument to evaluate patient satisfaction with care, which may clarify the impact of the intervention and the change in attitudes among healthcare providers. Future work could consider correlating healthcare provider attitudes with process and outcomes of care, including the length of stay.

## CONCLUSIONS

The opioid crisis is impacting healthcare practitioners in every sphere. The importance of changing the knowledge and attitudes of those in healthcare cannot be overstated. Judgmental attitudes of healthcare providers toward women with OUD can be a barrier to care.^23^ Women who feel judged are less likely to participate in their infant’s care, possibly impacting the length of stay, timeliness of discharge, and effectiveness of care.^6^ Education for the clinical team caring for the mother–infant dyad in trauma-informed care and substance use as a chronic disease, partnering with the mother of an infant with NAS and local community agencies, and most importantly, training in nonjudgmental care and attitude change, should be included in any initiative involving infants with NAS and their mothers. As the opioid epidemic overwhelms the nation, this attitudinal change is a critical component of effective obstetric and neonatal care.

## DISCLOSURE

The authors have no financial interest to declare in relation to the content of this article.

## Supplementary Material


