# Supplementary material for: Quality Improvement Initiative to Improve Healthcare Providers’ Attitudes towards Mothers with Opioid Use Disorder
Source: Pediatr Qual Saf. 2021 Aug 26;6(5):e453. doi: 10.1097/pq9.0000000000000453 (PMC8389895; doi:10.1097/pq9.0000000000000453)
Supplement: Supplementary file 1 [file pqs-6-e453-s001.pdf]

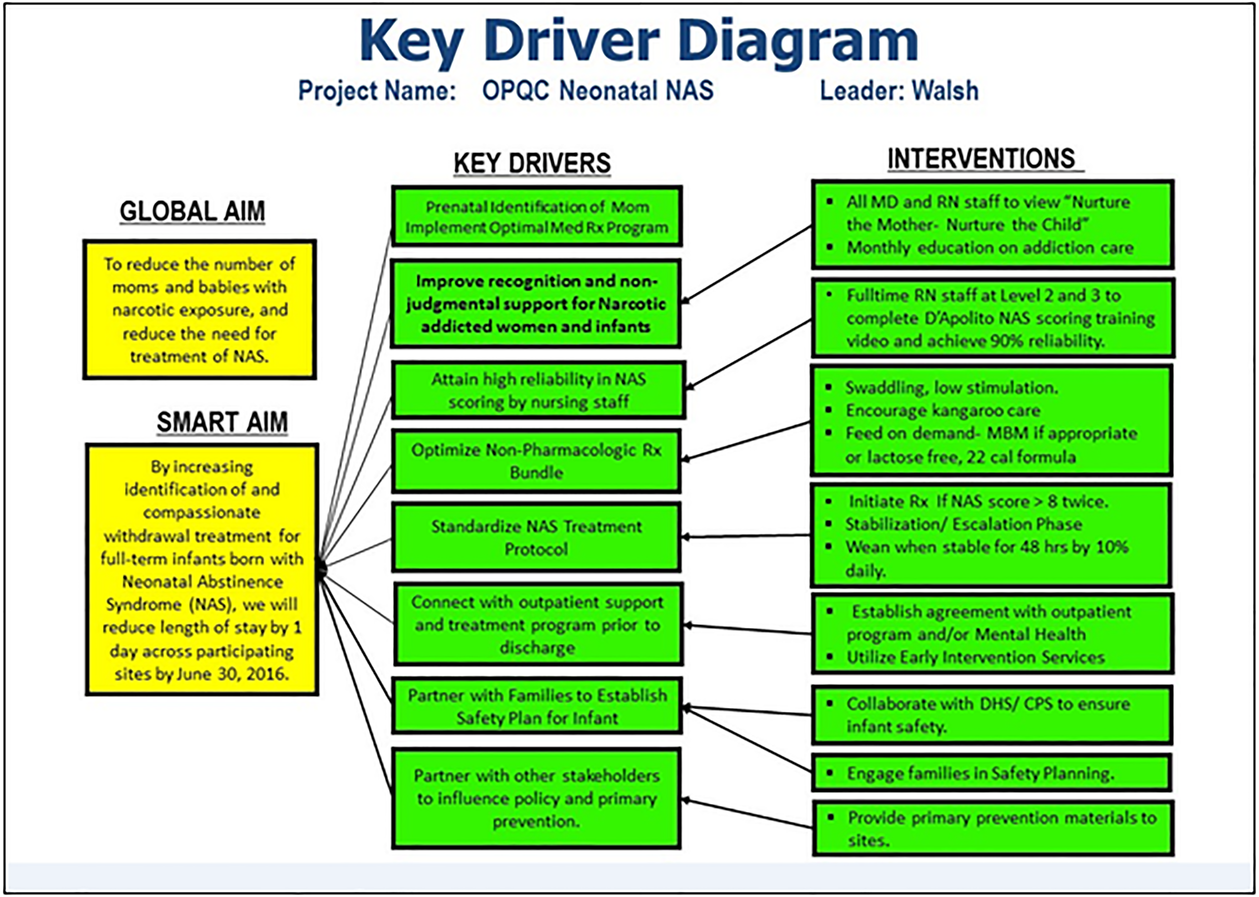
Key Driver Diagram
Project Name: OPQC Neonatal NAS
Leader: Walsh
GLOBAL AIM
To reduce the number of moms and babies with narcotic exposure, and reduce the need for treatment of NAS.
SMART AIM
By increasing identification of and compassionate withdrawal treatment for full-term infants born with Neonatal Abstinence Syndrome (NAS), we will reduce length of stay by 1 day across participating sites by June 30, 2016.
KEY DRIVERS
Prenatal Identification of Mom Implement Optimal Med Rx Program
Improve recognition and non-judgmental support for Narcotic addicted women and infants
Attain high reliability in NAS scoring by nursing staff
Optimize Non-Pharmacologic Rx Bundle
Standardize NAS Treatment Protocol
Connect with outpatient support and treatment program prior to discharge
Partner with Families to Establish Safety Plan for Infant
Partner with other stakeholders to influence policy and primary prevention.
INTERVENTIONS
• All MD and RN staff to view "Nurture the Mother- Nurture the Child"
• Monthly education on addiction care
• Fulltime RN staff at Level 2 and 3 to complete D'Apolito NAS scoring training video and achieve 90% reliability.
• Swaddling, low stimulation.
• Encourage kangaroo care
• Feed on demand- MBM if appropriate or lactose free, 22 cal formula
• Initiate Rx If NAS score > 8 twice.
• Stabilization/ Escalation Phase
• Wean when stable for 48 hrs by 10% daily.
• Establish agreement with outpatient program and/or Mental Health
• Utilize Early Intervention Services
• Collaborate with DHS/ CPS to ensure infant safety.
• Engage families in Safety Planning.
• Provide primary prevention materials to sites.
