# Supplementary material for: Quality Improvement Initiative to Improve Healthcare Providers’ Attitudes towards Mothers with Opioid Use Disorder
Source: Pediatr Qual Saf. 2021 Aug 26;6(5):e453. doi: 10.1097/pq9.0000000000000453 (PMC8389895; doi:10.1097/pq9.0000000000000453)
Supplement: Supplementary file 2 [file pqs-6-e453-s002.pdf]

SDC 2. NAS Project Action and Sustain Period Calls

Sustain Period Call Slides (2017 - 2018)

June 2018: NAS Project Accomplishments & Moving Upstream: MOMS+

March 2018: NAS Legislative Updates

December 2017: Alternative Sites of Care for NAS Infants

September 2017: NAS Care At and After Discharge

March 2017: Is Your Team Sustaining Your Gains?

Action Period Call Slides (2014 - 2016)

December 2016: Orchestrated Testing Data Analysis

November 2016: Sustainability - Do You Have a Plan?

October 2016: Overview from the Learning Session

July 2016: High Reliability of Formula Compliance

January 2016: The Year in Review - 2015

October 2015: Fall Learning Session Recap/Kick-off of NAS Phase-2

July 2015: Orchestrated Testing

June 2015: Onwards and Upwards: Next Steps for the NAS OPQC Project

May 2015: Improving Attitudes/MOMS Project Update

April 2015: Follow Up Care for NAS Infants

March 2015: Breastfeeding the NAS Infant -Challenges and Benefits

February 2015: Building on Collaboration from the Learning Session: Moving Forward

December 2014: Focus: Upcoming Learning Session

November 2014: Community Resources

October 2014: Educational Resources for Parents and Families Affected by Neonatal Abstinence Syndrome

September 2014: Legal and Practical Issues in Screening and Testing for Drug Use in Pregnancy

August 2014: Lessons Learned in Implementing the Pharmacological Bundle for Morphine or Methadone

July 2014: Applying Concepts from "The Improvement Guide" to work on the NAS Project

May 2014: The Pharmacological Bundle in the OPQC Protocol

April 2014: Completion of the Data Collection Tool; Definitions and Measures/Q&A

March 2014: Non-Pharmacological Treatment for the NAS Infant; guest presenter Dr. Bonny Whalen, Dartmouth Hitchcock Medical Center

February 2014: Data Collection Tool Testing/Journey to Improved NAS Scoring

Available at: https://www.opqc.net/nas-period-calls
