# Supplementary material for: Quality Improvement Initiative to Improve Healthcare Providers’ Attitudes towards Mothers with Opioid Use Disorder
Source: Pediatr Qual Saf. 2021 Aug 26;6(5):e453. doi: 10.1097/pq9.0000000000000453 (PMC8389895; doi:10.1097/pq9.0000000000000453)
Supplement: Supplementary file 3 [file pqs-6-e453-s003.pdf]

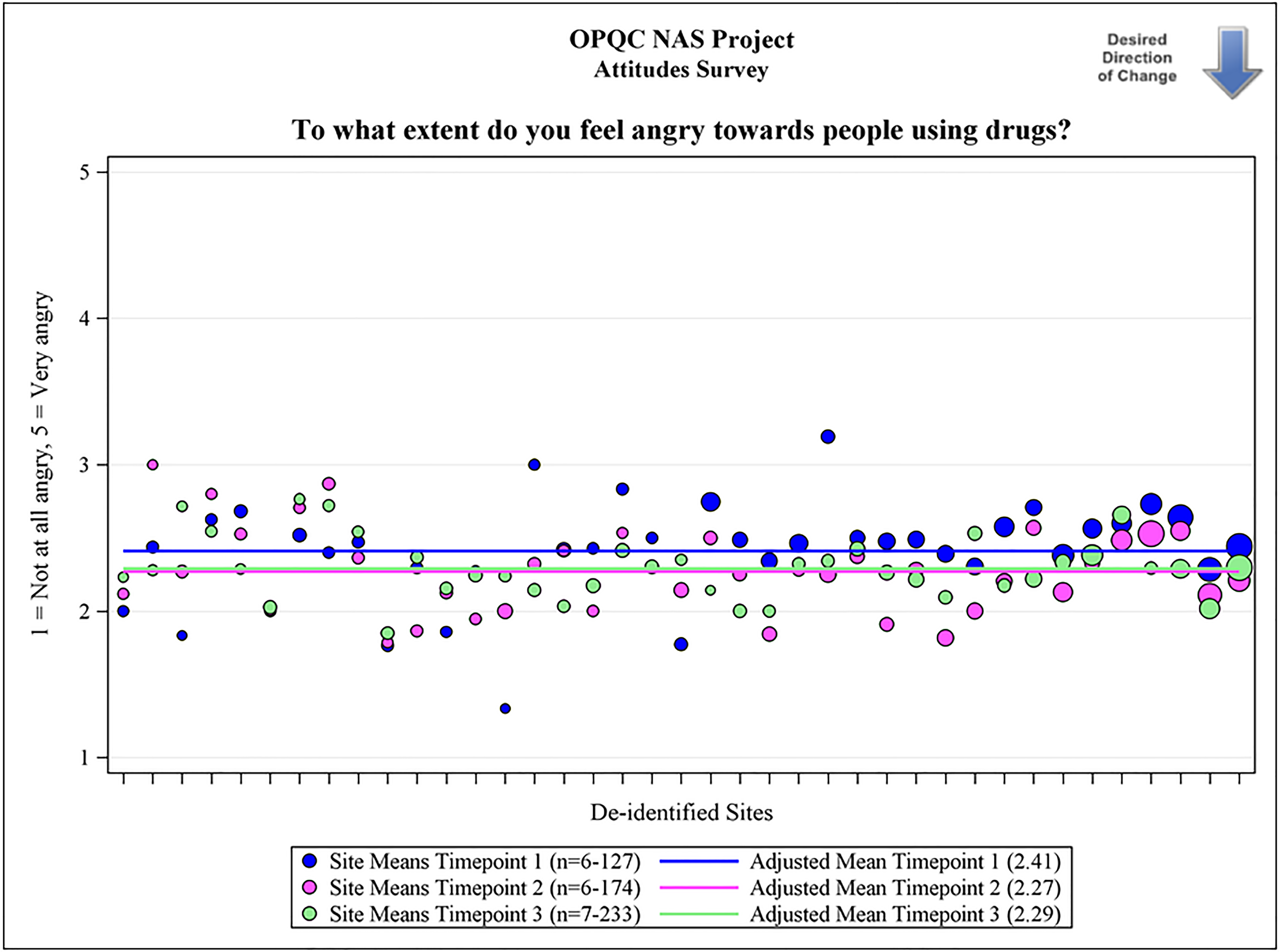

OPQC NAS Project
Attitudes Survey
Desired Direction of Change
To what extent do you feel angry towards people using drugs?
1 = Not at all angry, 5 = Very angry
5
4
3
2
1
De-identified Sites
Site Means Timepoint 1 (n=6-127)
Site Means Timepoint 2 (n=6-174)
Site Means Timepoint 3 (n=7-233)
Adjusted Mean Timepoint 1 (2.41)
Adjusted Mean Timepoint 2 (2.27)
Adjusted Mean Timepoint 3 (2.29)

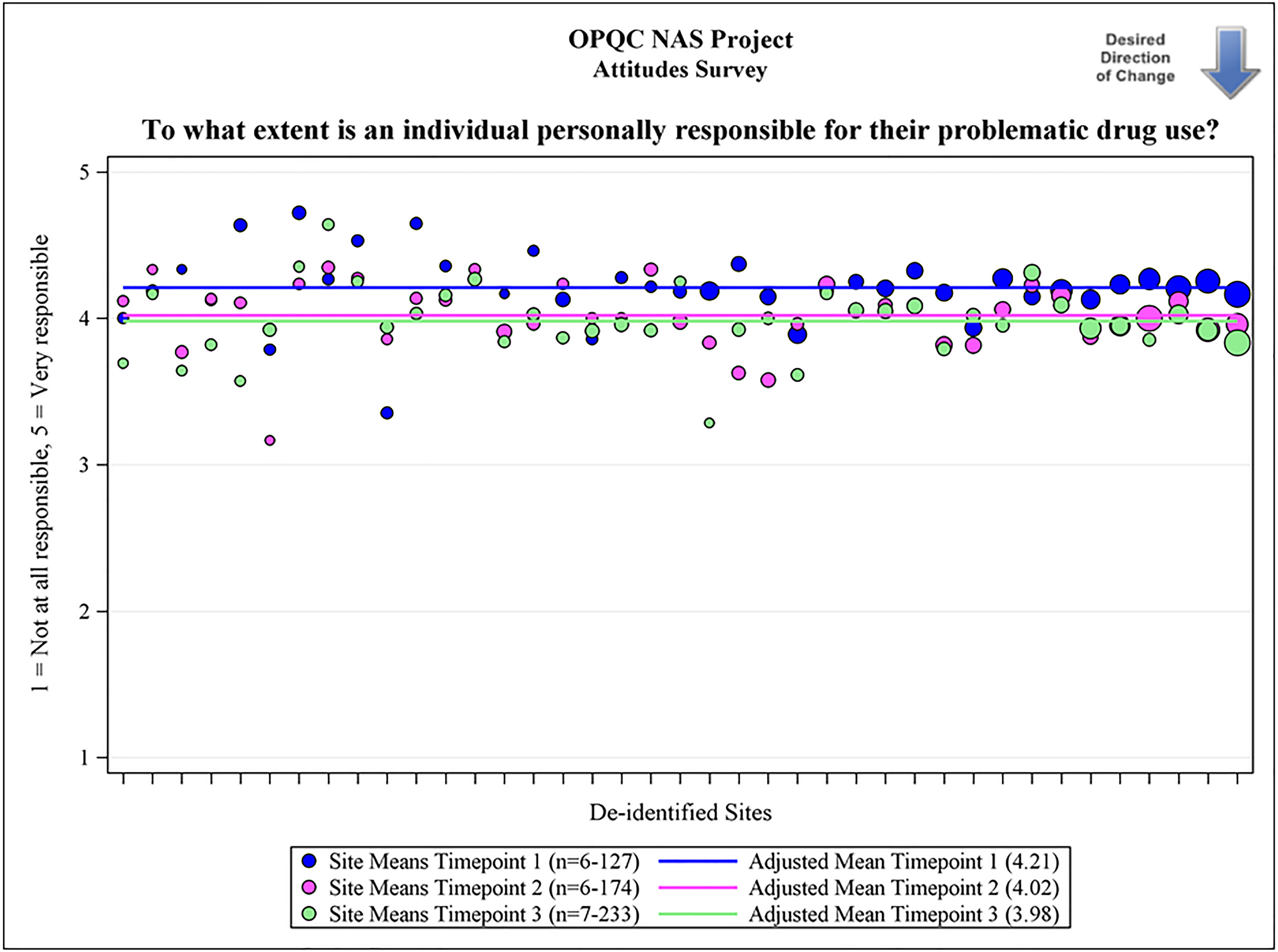

OPQC NAS Project
Attitudes Survey
Desired Direction of Change
To what extent is an individual personally responsible for their problematic drug use?
1 = Not at all responsible, 5 = Very responsible
5
4
3
2
1
De-identified Sites
Site Means Timepoint 1 (n=6-127)
Site Means Timepoint 2 (n=6-174)
Site Means Timepoint 3 (n=7-233)
Adjusted Mean Timepoint 1 (4.21)
Adjusted Mean Timepoint 2 (4.02)
Adjusted Mean Timepoint 3 (3.98)

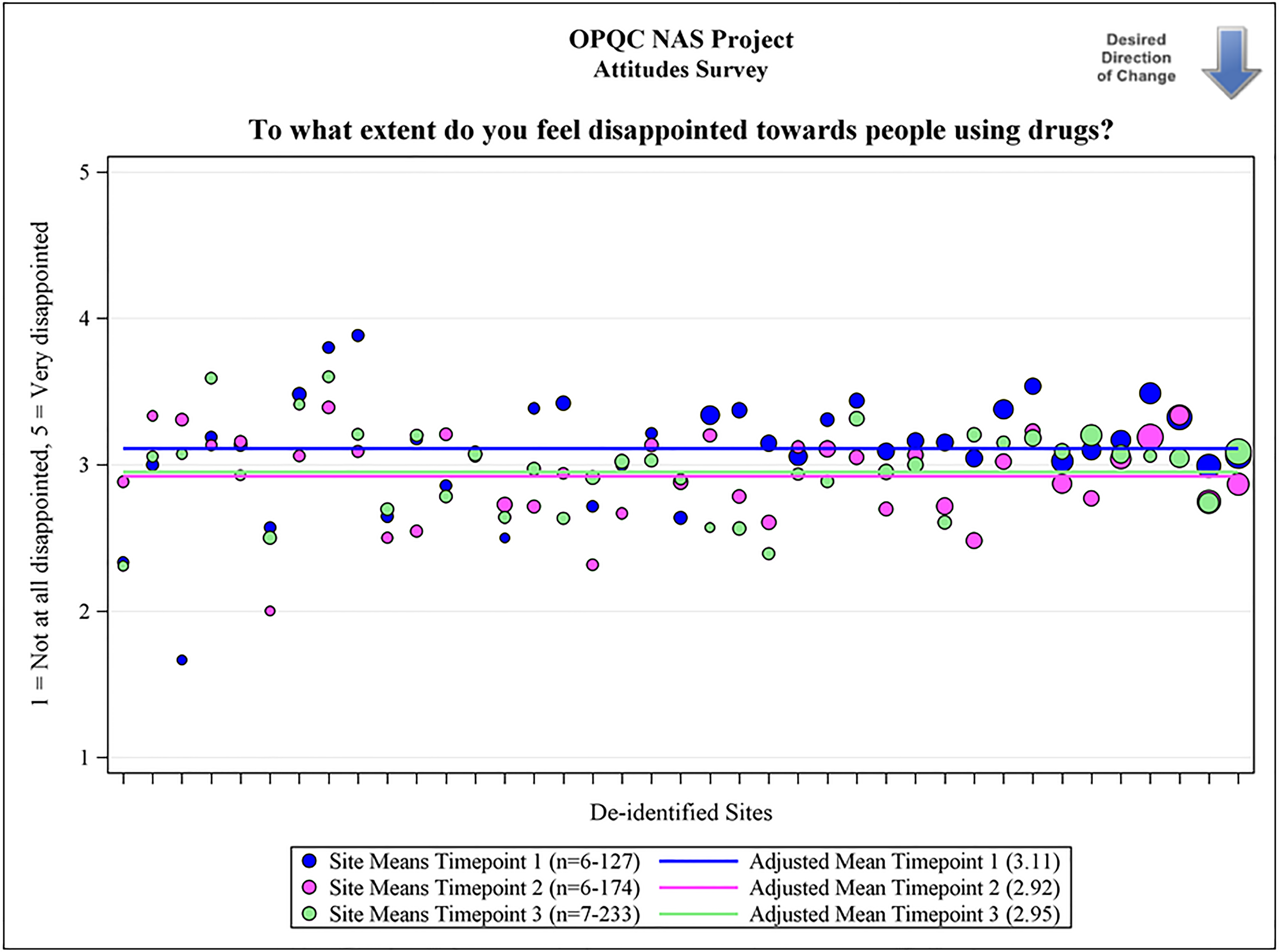

OPQC NAS Project
Attitudes Survey
Desired Direction of Change
To what extent do you feel disappointed towards people using drugs?
1 = Not at all disappointed, 5 = Very disappointed
5
4
3
2
1
De-identified Sites
Site Means Timepoint 1 (n=6-127)
Site Means Timepoint 2 (n=6-174)
Site Means Timepoint 3 (n=7-233)
Adjusted Mean Timepoint 1 (3.11)
Adjusted Mean Timepoint 2 (2.92)
Adjusted Mean Timepoint 3 (2.95)

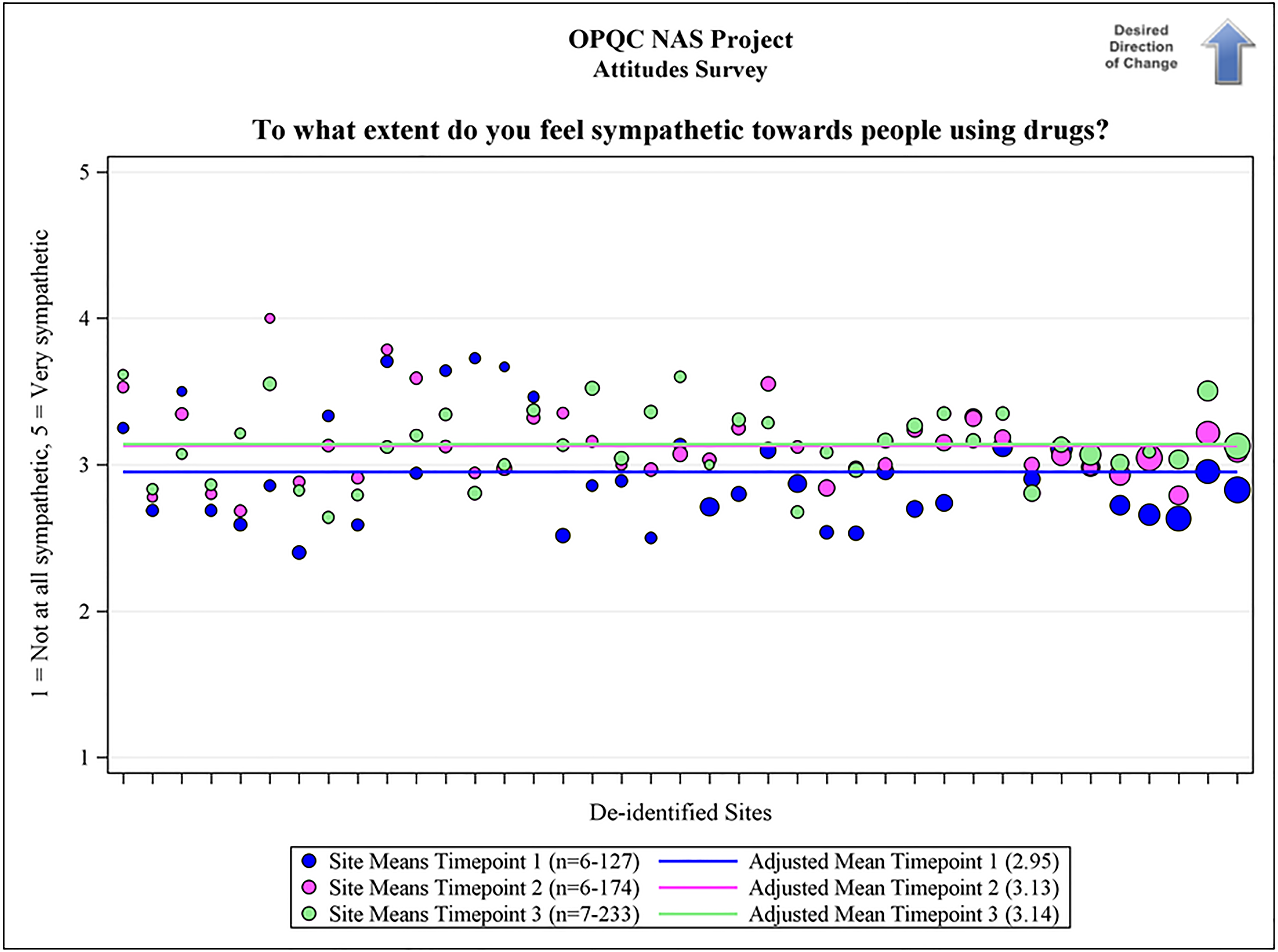
OPQC NAS Project
Attitudes Survey
Desired Direction of Change
To what extent do you feel sympathetic towards people using drugs?
1 = Not at all sympathetic, 5 = Very sympathetic
5
4
3
2
1
De-identified Sites
Site Means Timepoint 1 (n=6-127)
Site Means Timepoint 2 (n=6-174)
Site Means Timepoint 3 (n=7-233)
Adjusted Mean Timepoint 1 (2.95)
Adjusted Mean Timepoint 2 (3.13)
Adjusted Mean Timepoint 3 (3.14)

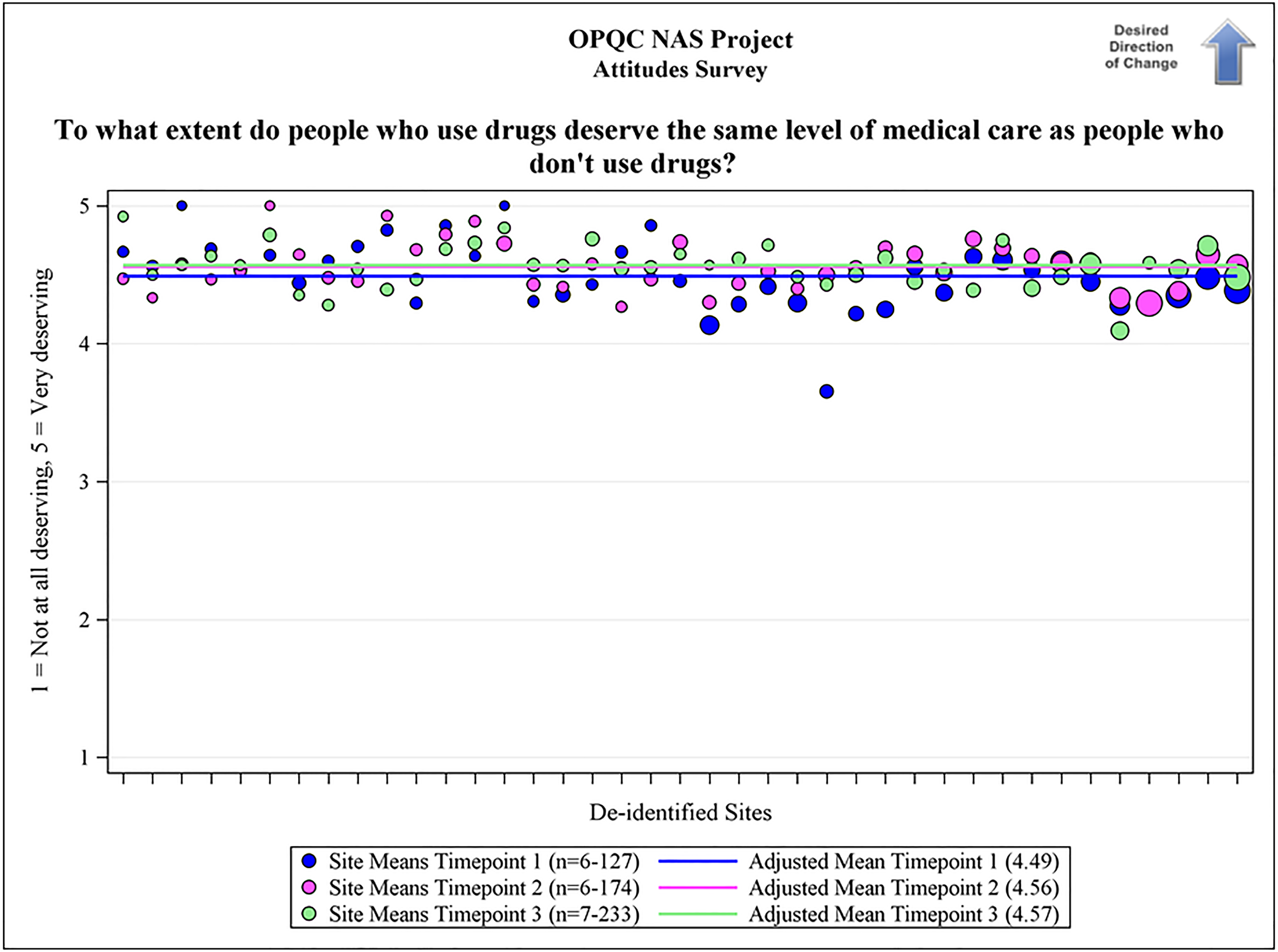
OPQC NAS Project
Attitudes Survey
Desired Direction of Change
To what extent do people who use drugs deserve the same level of medical care as people who don't use drugs?
1 = Not at all deserving, 5 = Very deserving
5
4
3
2
1
De-identified Sites
Site Means Timepoint 1 (n=6-127)
Site Means Timepoint 2 (n=6-174)
Site Means Timepoint 3 (n=7-233)
Adjusted Mean Timepoint 1 (4.49)
Adjusted Mean Timepoint 2 (4.56)
Adjusted Mean Timepoint 3 (4.57)
